# Supplementary material for: Consensus Definition and Prediction of Complexity in Transurethral Resection or Bladder Endoscopic Dissection of Bladder Tumours
Source: Cancers (Basel). 2020 Oct 20;12(10):3063. doi: 10.3390/cancers12103063 (PMC7589904; doi:10.3390/cancers12103063)
Supplement: Supplementary file 1 [file cancers-12-03063-s001.pdf]

Supplementary Materials: Consensus Definition and Prediction of Complexity in Transurethral Resection or Bladder Endoscopic Dissection of Bladder Tumours

Mathieu Roumiguié, Evanguelos Xylinas , Antonin Brisuda , Maximillian Burger , Hugh Mostafid, Marc Colombel, Marko Babjuk, Joan Palou Redorta, Fred Witjes and Bernard Malavaud

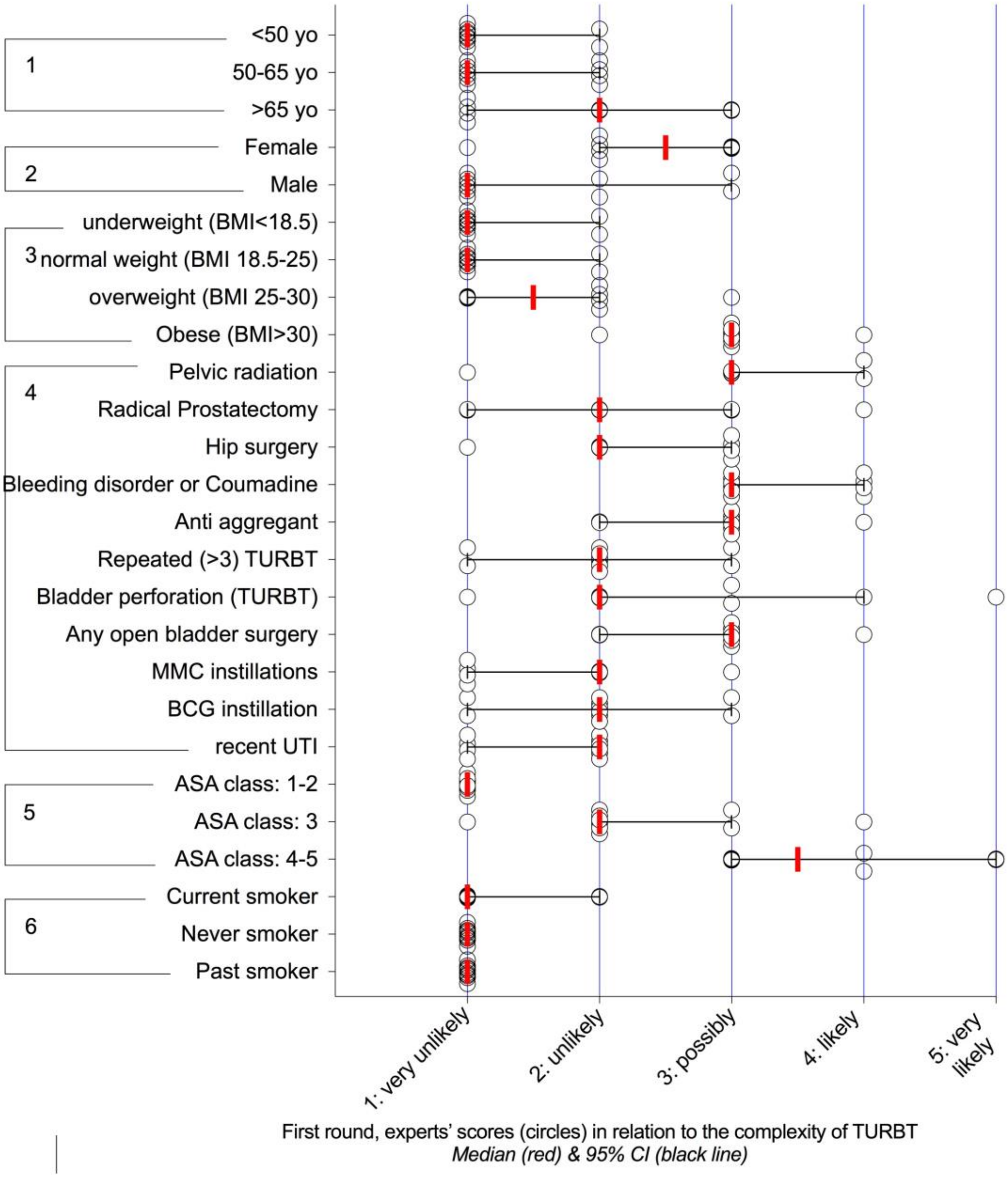

Supplementary Figure S1. First round distribution of the experts' scores regarding the influence of the characteristics of the patient on the likelihood of complex TURBT.(1) age, (2) sex, (3) weight & body mass index (BMI), (4) patient's history, (5) American Society of Anesthesiologists' physical status classification (ASA), (6) tobacco smoking.

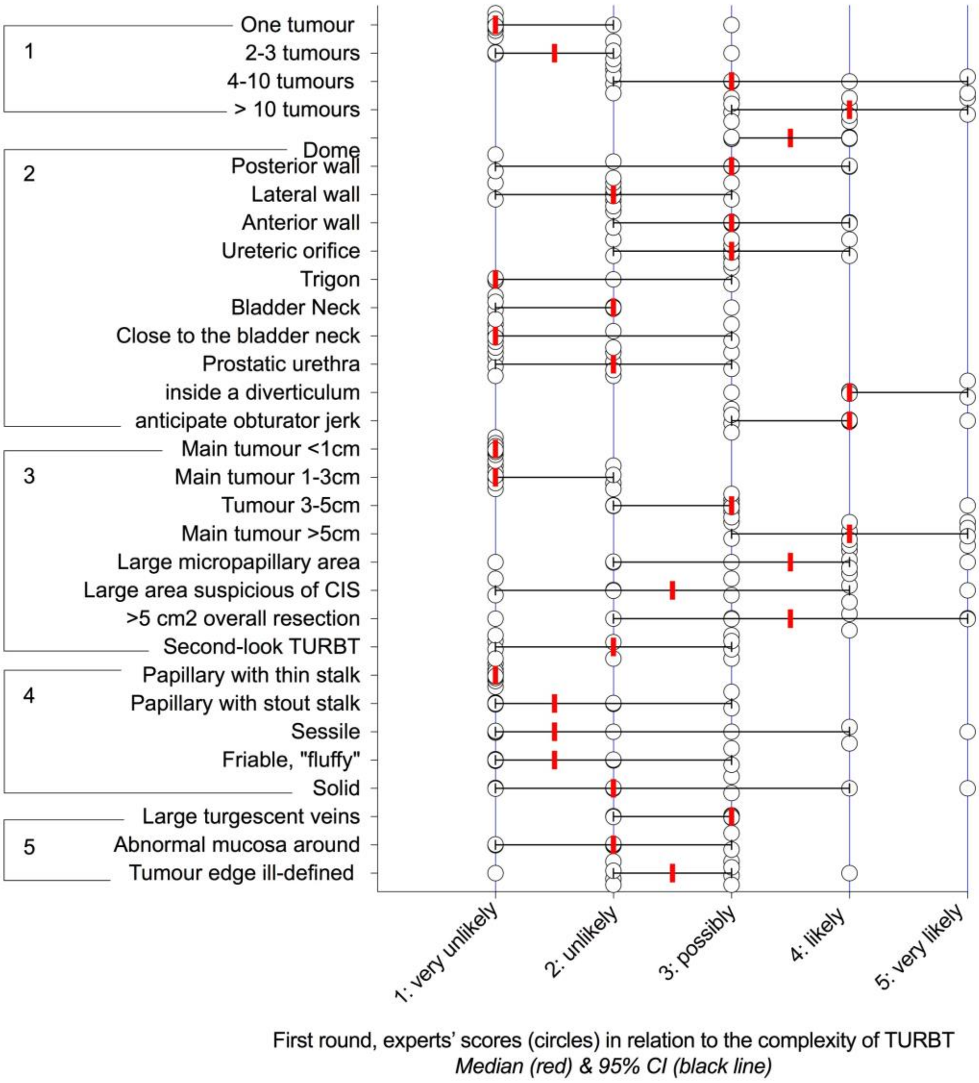

Supplementary Figure S2. First round distribution of the experts' scores regarding the influence of the characteristics of the tumour on the likelihood of complex TURBT. (1) number, (2) location, (3) size, (4) structure, (5) environment.

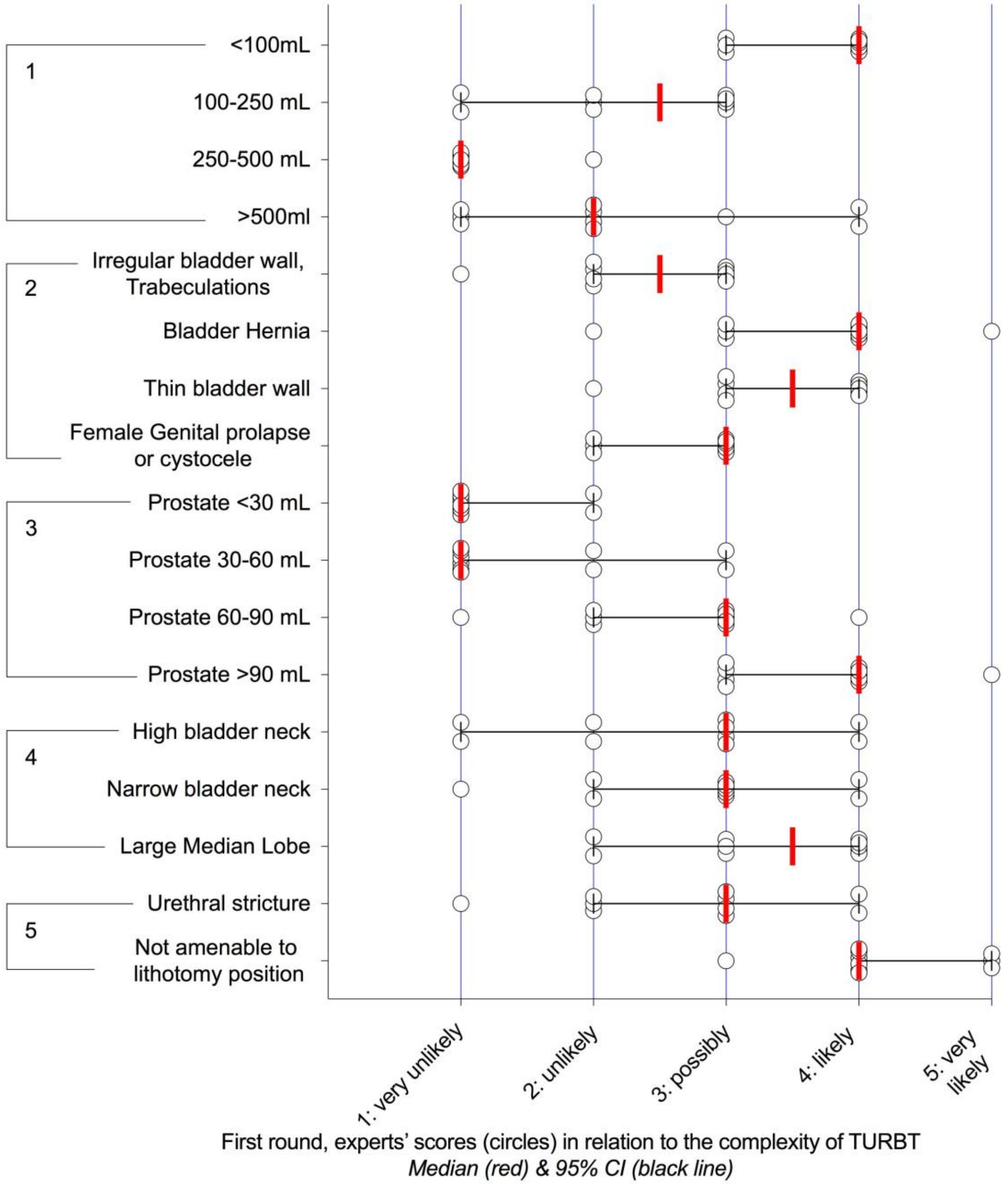

Supplementary Figure S3. First round distribution of the experts' scores regarding the influence on the likelihood of complex TURBT of bladder characteristics and access. (1) bladder capacity, (2) bladder structure, (3) prostate volume, (4) bladder neck, (5) other.

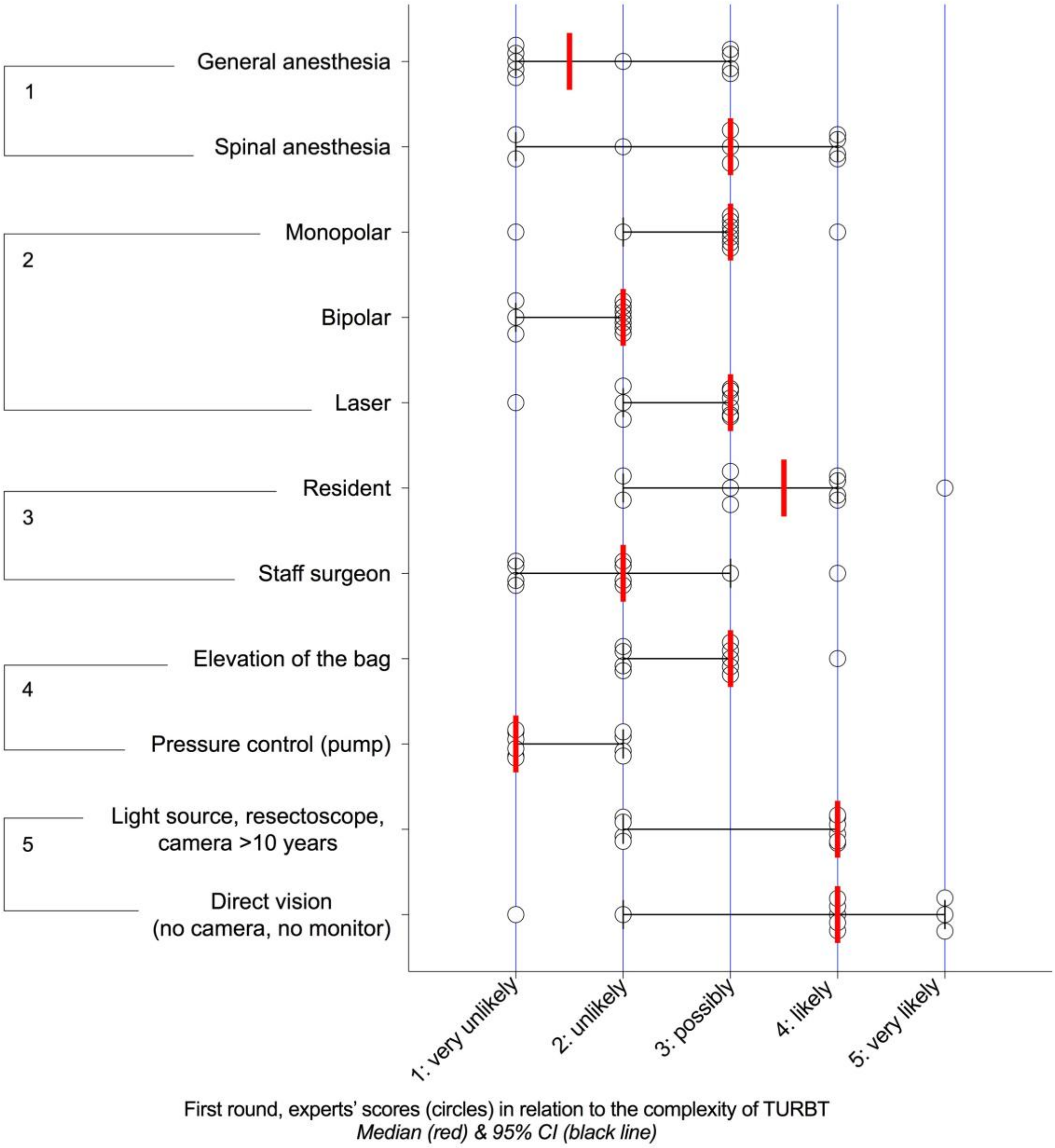

Supplementary Figure S4. First round distribution of the experts' scores regarding the influence of the surgical environment on the risk of TURBT or En-Bloc resection resulting in either three situations; incomplete resection according to the operator, or prolonged surgery (>1hour) or significant intra (bleeding that requires transfusion, laparotomy) or postoperative (Clavien-Dindo Grade III and higher) (1) Anaesthesia, (2) energy, (3) operator, (4) bladder irrigation, (5) instruments.

Supplementary Table 1. Articles (English language, 4/2009-4/2019) found relevant to the definition of complexity in transurethral resection of bladder tumours.

First Author [reference]	Year	Adequacy*	Intraoperative Events **	Postoperative Events
Mariappan [9]	2010	+		
Gan [10]	2013	+		
Prasad [11]	2017	+		
Skrzypczyk [12]	2017	+		
DelRosso [13]	2013	+	+	
Pan [14]	2012	+	+	+
Venkatramani [15]	2013	+	+	+
Wu [16]	2016	+	+	+
Zhang [17]	2017		+	
Herkommer [18]	2012		+	

Golan [19]	2011	+	+
Carmignani [20]	2011	+	+
Zhao [21]	2016	+	+
Sugihara [22]	2014	+	+
Allard [23]	2015	+	+
Patel [24]	2015	+	+
Ghali [8]	2016	+	+
Avallone [25]	2017	+	+
Rambachan [26]	2014		+
Matulewicz [27]	2014		+
Picozzi [28]	2014		+
DeNunzio [29]	2014		+
Valerio [30]	2014		+
Matulewicz [31]	2015		+
DiPaolo [32]	2015		+
Gregg [33]	2016		+
Cornu [34]	2016		+
Bolat [35]	2016		+
Bansal [36]	2016		+
Anderson [37]	2016		+
Konishi [38]	2017		+
Prader [39]	2017		+
Caras [40]	2017		+
Naspro [41]	2018		+
Suskind [42]	2018		+
Pereira [4]	2019		+

*Adequacy refers to the quality of the resected specimen, including visually complete resection and presence of muscle at the resection base.

** Intraoperative events, including operative time.

Supplementary Table S2: 150 scenarios constructed for univariate and multivariate analyses of clinical features in relation to complexity.

List B	Age	Sex	Patient’s History	Tumour Number	Main Tumour location	Main Tumour size	Main Tumour structure	Bladder anatomy	Access
1	>65 yo	Male	Hx of bleeding disorder or	4 to 10 lesions	Trigone	>5cm	with large turgescent veins	large bladder (250-500mL)	not amenable to the lithotomy position
2	>65 yo	Male	Hx of bleeding disorder or	2 to 3 lesions	Lateral wall	1-3cm	papillary with a stout, large pedicle	very large bladder (>500mL)	not amenable to the lithotomy position
3	<50 yo	Female	Hx of hip surgery	2 to 3 lesions	Trigone	resection of a TURBT scar (e.g “second look	with abnormal mucosa extending beyond	small bladder (<100mL)	no relevant features
4	<50 yo	Female	Hx of bleeding disorder or	over 10 lesions	Lateral wall	1-3cm	with abnormal mucosa extending beyond	small bladder (<100mL)	not amenable to the lithotomy position
5	50-65 yo	Male	no relevant history	2 to 3 lesions	Anterior wall	< 1cm	Sessile or solid aspect	large bladder (250-500mL)	large median lobe
6	<50 yo	Female	Female genital prolapse o	Single lesion	Ureteric orifice	resection of a TURBT scar (e.g “second look	ill-defined tumour limits	thin bladder wall	no relevant features
7	<50 yo	Male	no relevant history	2 to 3 lesions	Ureteric orifice	1-3cm	Sessile or solid aspect	very large bladder (>500mL)	mid-size prostate (30-60mL)
8	50-65 yo	Female	no relevant history	Single lesion	Ureteric orifice	Large micropapillary area (>5cm2)	ill-defined tumour limits	large bladder (250-500mL)	narrow bladder neck
9	<50 yo	Male	no relevant history	Single lesion	Ureteric orifice	>5cm	Sessile or solid aspect	very large bladder (>500mL)	large median lobe
10	50-65 yo	Male	Hx of recent (<3 months)	4 to 10 lesions	inside a diverticulum	1-3cm	papillary with a stout, large pedicle	normal bladder (100-250mL)	Narrow bladder neck
11	<50 yo	Male	no relevant history	Single lesion	Ureteric orifice	>5cm	Sessile or solid aspect	normal bladder (100-250mL)	Narrow bladder neck
12	>65 yo	Male	Hx of Bladder perforation	Single lesion	Lateral wall	>5cm	Sessile or solid aspect	bladder hernia	large median lobe
13	50-65 yo	Male	no relevant history	2 to 3 lesions	Bladder neck	>5cm	papillary with a thin pedicle	small bladder (<100mL)	mid-size prostate (30-60mL)
14	>65 yo	Female	no relevant history	over 10 lesions	Ureteric orifice	3-5cm	Friable, “fluffy” aspect	thin bladder wall	no relevant features
15	50-65 yo	Male	ASA class: 4-5	Single lesion	Prostatic urethra	1-3cm	ill-defined tumour limits	very large bladder (>500mL)	Narrow bladder neck
16	>65 yo	Male	Hx of recent (<3 months)	4 to 10 lesions	Trigone	< 1cm	papillary with a stout, large pedicle	thin bladder wall	mid-size prostate (30-60mL)
17	<50 yo	Female	ASA class: 4-5	over 10 lesions	Dome	1-3cm	with abnormal mucosa extending beyond	very large bladder (>500mL)	no relevant features
18	<50 yo	Male	Hx of pelvic radiation	Single lesion	inside a diverticulum	1-3cm	ill-defined tumour limits	very large bladder (>500mL)	Narrow bladder neck
19	50-65 yo	Male	no relevant history	Single lesion	Posterior wall	>5cm	papillary with a thin pedicle	irregular bladder wall, trabeculations	small prostate (<30mL)
20	<50 yo	Male	no relevant history	Single lesion	anticipate obturator jerk	< 1cm	with large turgescent veins	irregular bladder wall, trabeculations	Narrow bladder neck
21	<50 yo	Male	no relevant history	over 10 lesions	Prostatic urethra	>5cm	papillary with a thin pedicle	normal bladder (100-250mL)	small prostate (<30mL)
22	50-65 yo	Female	Hx of antiplatelet treatme	Single lesion	Anterior wall	< 1cm	ill-defined tumour limits	very large bladder (>500mL)	no relevant features
23	50-65 yo	Female	Hx of hip surgery	over 10 lesions	Anterior wall	3-5cm	with abnormal mucosa extending beyond	very large bladder (>500mL)	no relevant features
24	<50 yo	Male	no relevant history	over 10 lesions	Ureteric orifice	>5cm	with abnormal mucosa extending beyond	small bladder (<100mL)	Urethral stricture
25	>65 yo	Male	Hx of prostatectomy	over 10 lesions	Posterior wall	1-3cm	with abnormal mucosa extending beyond	large bladder (250-500mL)	no relevant features
26	<50 yo	Male	ASA class: 4-5	4 to 10 lesions	Bladder neck	resection of a TURBT scar (e.g “second look	ill-defined tumour limits	large bladder (250-500mL)	large prostate (60-90mL)
27	>65 yo	Male	no relevant history	4 to 10 lesions	anticipate obturator jerk	3-5cm	ill-defined tumour limits	large bladder (250-500mL)	mid-size prostate (30-60mL)
28	50-65 yo	Male	no relevant history	Single lesion	Dome	>5cm	Sessile or solid aspect	large bladder (250-500mL)	very large prostate (>90mL)
29	<50 yo	Female	no relevant history	Single lesion	Lateral wall	1-3cm	with large turgescent veins	normal bladder (100-250mL)	no relevant features
30	50-65 yo	Female	no relevant history	over 10 lesions	Ureteric orifice	1-3cm	with large turgescent veins	normal bladder (100-250mL)	narrow bladder neck
31	50-65 yo	Female	no relevant history	over 10 lesions	Anterior wall	1-3cm	Sessile or solid aspect	small bladder (<100mL)	no relevant features
32	>65 yo	Female	no relevant history	over 10 lesions	Dome	>5cm	with large turgescent veins	thin bladder wall	not amenable to the lithotomy position
33	50-65 yo	Male	Antiplatelet treatment	Single lesion	Anterior wall	resection of a TURBT scar (e.g “second look	with abnormal mucosa extending beyond	normal bladder (100-250mL)	small prostate (<30mL)
34	>65 yo	Male	Bladder surgery (reimplan	2 to 3 lesions	Lateral wall	resection of a TURBT scar (e.g “second look	ill-defined tumour limits	irregular bladder wall, trabeculations	Narrow bladder neck
35	50-65 yo	Male	no relevant history	4 to 10 lesions	Lateral wall	Large micropapillary area (>5cm2)	ill-defined tumour limits	large bladder (250-500mL)	Narrow bladder neck
36	50-65 yo	Male	no relevant history	over 10 lesions	Anterior wall	3-5cm	with large turgescent veins	large bladder (250-500mL)	Narrow bladder neck
37	50-65 yo	Male	Hx of repeated (>3) TURB	over 10 lesions	Anterior wall	< 1cm	with abnormal mucosa extending beyond	normal bladder (100-250mL)	Narrow bladder neck
38	50-65 yo	Male	no relevant history	4 to 10 lesions	Lateral wall	Large micropapillary area (>5cm2)	with large turgescent veins	large bladder (250-500mL)	small prostate (<30mL)
39	>65 yo	Male	no relevant history	Single lesion	Dome	Large denudated area suspicious of CIS (>5c	with abnormal mucosa extending beyond	large bladder (250-500mL)	not amenable to the lithotomy position
40	50-65 yo	Male	no relevant history	Single lesion	Ureteric orifice	1-3cm	ill-defined tumour limits	very large bladder (>500mL)	small prostate (<30mL)
41	<50 yo	Female	no relevant history	Single lesion	Anterior wall	>5cm	papillary with a stout, large pedicle	small bladder (<100mL)	no relevant features
42	<50 yo	Male	Bladder surgery (reimplan	2 to 3 lesions	Bladder neck	Large denudated area suspicious of CIS (>5c	with abnormal mucosa extending beyond	bladder hernia	very large prostate (>90mL)
43	50-65 yo	Male	no relevant history	2 to 3 lesions	Trigone	< 1cm	Sessile or solid aspect	normal bladder (100-250mL)	Urethral stricture
44	>65 yo	Male	no relevant history	2 to 3 lesions	Lateral wall	Large denudated area suspicious of CIS (>5c	with large turgescent veins	bladder hernia	small prostate (<30mL)
45	>65 yo	Male	no relevant history	2 to 3 lesions	Bladder neck	1-3cm	ill-defined tumour limits	very large bladder (>500mL)	small prostate (<30mL)
46	>65 yo	Male	no relevant history	over 10 lesions	Posterior wall	>5cm	with large turgescent veins	normal bladder (100-250mL)	Urethral stricture
47	<50 yo	Female	Obese (BMI>30)	2 to 3 lesions	Dome	3-5cm	papillary with a stout, large pedicle	thin bladder wall	urethral stricture
48	<50 yo	Male	Bladder surgery (reimplan	2 to 3 lesions	Dome	1-3cm	papillary with a stout, large pedicle	large bladder (250-500mL)	large median lobe
49	50-65 yo	Female	no relevant history	Single lesion	Trigone	Large denudated area suspicious of CIS (>5c	ill-defined tumour limits	thin bladder wall	narrow bladder neck
50	50-65 yo	Male	Bladder surgery (reimplan	Single lesion	inside a diverticulum	Large micropapillary area (>5cm2)	with abnormal mucosa extending beyond	bladder hernia	Narrow bladder neck
51	>65 yo	Female	Obese (BMI>30)	over 10 lesions	Dome	Large denudated area suspicious of CIS (>5c	ill-defined tumour limits	small bladder (<100mL)	no relevant features
52	50-65 yo	Male	no relevant history	2 to 3 lesions	Posterior wall	< 1cm	with abnormal mucosa extending beyond	large bladder (250-500mL)	large prostate (60-90mL)
53	<50 yo	Male	no relevant history	over 10 lesions	Bladder neck	>5cm	Sessile or solid aspect	very large bladder (>500mL)	Urethral stricture
54	<50 yo	Female	Hx of Bladder perforation	Single lesion	Posterior wall	Large denudated area suspicious of CIS (>5c	ill-defined tumour limits	bladder hernia	not amenable to the lithotomy position
55	>65 yo	Male	Hx of bleeding disorder or	Single lesion	Lateral wall	1-3cm	papillary with a thin pedicle	thin bladder wall	Narrow bladder neck
56	50-65 yo	Male	Hx of pelvic radiation	2 to 3 lesions	Bladder neck	Large micropapillary area (>5cm2)	with large turgescent veins	small bladder (<100mL)	small prostate (<30mL)
57	<50 yo	Female	Bladder surgery (reimplan	Single lesion	Trigone	>5cm	with large turgescent veins	very large bladder (>500mL)	no relevant features
58	50-65 yo	Female	Hx of hip surgery	4 to 10 lesions	Trigone	>5cm	Friable, “fluffy” aspect	small bladder (<100mL)	not amenable to the lithotomy position
59	<50 yo	Male	no relevant history	Single lesion	Bladder neck	< 1cm	Sessile or solid aspect	normal bladder (100-250mL)	very large prostate (>90mL)
60	>65 yo	Male	Bladder surgery (reimplan	Single lesion	Dome	resection of a TURBT scar (e.g “second look	with large turgescent veins	large bladder (250-500mL)	large median lobe
61	50-65 yo	Male	no relevant history	4 to 10 lesions	Dome	3-5cm	with abnormal mucosa extending beyond	very large bladder (>500mL)	not amenable to the lithotomy position
62	<50 yo	Male	no relevant history	Single lesion	Anterior wall	>5cm	with large turgescent veins	normal bladder (100-250mL)	large prostate (60-90mL)
63	<50 yo	Male	Antiplatelet treatment	2 to 3 lesions	inside a diverticulum	3-5cm	Sessile or solid aspect	normal bladder (100-250mL)	large prostate (60-90mL)
64	50-65 yo	Male	Antiplatelet treatment	over 10 lesions	Trigone	resection of a TURBT scar (e.g “second look	with large turgescent veins	small bladder (<100mL)	not amenable to the lithotomy position
65	>65 yo	Female	Hx of Bladder perforation	Single lesion	Bladder neck	< 1cm	Friable, “fluffy” aspect	thin bladder wall	not amenable to the lithotomy position
66	>65 yo	Male	Hx of intravesical chemot	4 to 10 lesions	Trigone	Large micropapillary area (>5cm2)	with abnormal mucosa extending beyond	small bladder (<100mL)	Narrow bladder neck
67	<50 yo	Male	no relevant history	2 to 3 lesions	Dome	resection of a TURBT scar (e.g “second look	with abnormal mucosa extending beyond	normal bladder (100-250mL)	very large prostate (>90mL)
68	50-65 yo	Female	no relevant history	over 10 lesions	Dome	3-5cm	Friable, “fluffy” aspect	normal bladder (100-250mL)	not amenable to the lithotomy position
69	>65 yo	Male	no relevant history	4 to 10 lesions	Lateral wall	>5cm	papillary with a thin pedicle	irregular bladder wall, trabeculations	small prostate (<30mL)
70	>65 yo	Male	no relevant history	2 to 3 lesions	Anterior wall	3-5cm	papillary with a stout, large pedicle	thin bladder wall	Urethral stricture
71	>65 yo	Female	Hx of hip surgery	Single lesion	anticipate obturator jerk	3-5cm	Friable, “fluffy” aspect	bladder hernia	narrow bladder neck
72	50-65 yo	Male	no relevant history	4 to 10 lesions	anticipate obturator jerk	Large micropapillary area (>5cm2)	ill-defined tumour limits	bladder hernia	small prostate (<30mL)
73	50-65 yo	Male	Antiplatelet treatment	Single lesion	Ureteric orifice	>5cm	ill-defined tumour limits	bladder hernia	large median lobe
74	<50 yo	Male	no relevant history	4 to 10 lesions	Posterior wall	>5cm	ill-defined tumour limits	normal bladder (100-250mL)	mid-size prostate (30-60mL)
75	>65 yo	Female	Obese (BMI>30)	Single lesion	Lateral wall	3-5cm	Sessile or solid aspect	very large bladder (>500mL)	no relevant features
76	>65 yo	Female	no relevant history	4 to 10 lesions	Posterior wall	1-3cm	papillary with a stout, large pedicle	normal bladder (100-250mL)	narrow bladder neck
77	>65 yo	Female	Obese (BMI>30)	2 to 3 lesions	Ureteric orifice	< 1cm	papillary with a stout, large pedicle	bladder hernia	not amenable to the lithotomy position
78	50-65 yo	Female	Obese (BMI>30)	4 to 10 lesions	Ureteric orifice	>5cm	Sessile or solid aspect	large bladder (250-500mL)	no relevant features
79	>65 yo	Male	Bladder surgery (reimplan	2 to 3 lesions	inside a diverticulum	< 1cm	Sessile or solid aspect	thin bladder wall	large median lobe
80	>65 yo	Female	no relevant history	Single lesion	inside a diverticulum	< 1cm	ill-defined tumour limits	thin bladder wall	urethral stricture
81	>65 yo	Male	no relevant history	4 to 10 lesions	anticipate obturator jerk	>5cm	papillary with a thin pedicle	large bladder (250-500mL)	Urethral stricture
82	>65 yo	Male	no relevant history	2 to 3 lesions	Dome	1-3cm	papillary with a stout, large pedicle	normal bladder (100-250mL)	large median lobe
83	<50 yo	Female	Hx of antiplatelet treatme	4 to 10 lesions	Anterior wall	Large micropapillary area (>5cm2)	ill-defined tumour limits	normal bladder (100-250mL)	no relevant features
84	50-65 yo	Female	no relevant history	over 10 lesions	anticipate obturator jerk	< 1cm	papillary with a stout, large pedicle	bladder hernia	not amenable to the lithotomy position
85	<50 yo	Male	Bladder surgery (reimplan	over 10 lesions	anticipate obturator jerk	< 1cm	Sessile or solid aspect	normal bladder (100-250mL)	Urethral stricture
86	>65 yo	Male	no relevant history	Single lesion	Posterior wall	3-5cm	papillary with a stout, large pedicle	bladder hernia	large median lobe
87	50-65 yo	Male	no relevant history	over 10 lesions	Dome	Large micropapillary area (>5cm2)	with large turgescent veins	large bladder (250-500mL)	small prostate (<30mL)
88	>65 yo	Female	ASA class: 4-5	Single lesion	Dome	>5cm	papillary with a stout, large pedicle	very large bladder (>500mL)	no relevant features
89	>65 yo	Male	no relevant history	4 to 10 lesions	Bladder neck	Large micropapillary area (>5cm2)	with abnormal mucosa extending beyond	normal bladder (100-250mL)	very large prostate (>90mL)
90	<50 yo	Male	Hx of intravesical BCG	2 to 3 lesions	Lateral wall	< 1cm	with abnormal mucosa extending beyond	small bladder (<100mL)	mid-size prostate (30-60mL)
91	50-65 yo	Female	Hx of intravesical BCG	2 to 3 lesions	inside a diverticulum	3-5cm	with large turgescent veins	normal bladder (100-250mL)	no relevant features
92	50-65 yo	Male	Antiplatelet treatment	4 to 10 lesions	Bladder neck	>5cm	papillary with a stout, large pedicle	large bladder (250-500mL)	very large prostate (>90mL)
93	>65 yo	Female	no relevant history	2 to 3 lesions	Posterior wall	Large denudated area suspicious of CIS (>5c	ill-defined tumour limits	normal bladder (100-250mL)	no relevant features
94	50-65 yo	Female	Hx of pelvic radiation	Single lesion	Trigone	Large denudated area suspicious of CIS (>5c	ill-defined tumour limits	normal bladder (100-250mL)	narrow bladder neck
95	50-65 yo	Male	no relevant history	4 to 10 lesions	Bladder neck	>5cm	with large turgescent veins	bladder hernia	not amenable to the lithotomy position
96	50-65 yo	Male	Antiplatelet treatment	over 10 lesions	Lateral wall	Large micropapillary area (>5cm2)	with abnormal mucosa extending beyond	very large bladder (>500mL)	mid-size prostate (30-60mL)
97	>65 yo	Female	Hx of hip surgery	over 10 lesions	Bladder neck	Large micropapillary area (>5cm2)	ill-defined tumour limits	large bladder (250-500mL)	no relevant features
98	<50 yo	Male	ASA class: 4-5	Single lesion	anticipate obturator jerk	>5cm	Sessile or solid aspect	normal bladder (100-250mL)	large prostate (60-90mL)
99	<50 yo	Female	Hx of intravesical BCG	2 to 3 lesions	Dome	< 1cm	papillary with a thin pedicle	very large bladder (>500mL)	no relevant features
100	>65 yo	Male	no relevant history	4 to 10 lesions	Trigone	Large denudated area suspicious of CIS (>5c	ill-defined tumour limits	large bladder (250-500mL)	small prostate (<30mL)
101	<50 yo	Male	no relevant history	4 to 10 lesions	Ureteric orifice	resection of a TURBT scar (e.g “second look	with abnormal mucosa extending beyond	large bladder (250-500mL)	Narrow bladder neck
102	<50 yo	Male	Hx of bleeding disorder or	Single lesion	Posterior wall	3-5cm	ill-defined tumour limits	small bladder (<100mL)	small prostate (<30mL)
103	<50 yo	Female	no relevant history	4 to 10 lesions	Dome	< 1cm	papillary with a thin pedicle	normal bladder (100-250mL)	no relevant features
104	>65 yo	Male	Bladder surgery (reimplan	over 10 lesions	anticipate obturator jerk	1-3cm	Sessile or solid aspect	large bladder (250-500mL)	mid-size prostate (30-60mL)
105	<50 yo	Male	Obese (BMI>30)	over 10 lesions	Prostatic urethra	Large denudated area suspicious of CIS (>5c	with large turgescent veins	large bladder (250-500mL)	mid-size prostate (30-60mL)
106	50-65 yo	Female	no relevant history	2 to 3 lesions	Ureteric orifice	3-5cm	papillary with a stout, large pedicle	large bladder (250-500mL)	no relevant features
107	50-65 yo	Female	Hx of recent (<3 months)	4 to 10 lesions	Posterior wall	resection of a TURBT scar (e.g “second look	with large turgescent veins	bladder hernia	no relevant features
108	<50 yo	Female	Bladder surgery (reimplan	over 10 lesions	Anterior wall	Large denudated area suspicious of CIS (>5c	ill-defined tumour limits	very large bladder (>500mL)	no relevant features
109	50-65 yo	Female	Hx of recent (<3 months)	2 to 3 lesions	anticipate obturator jerk	3-5cm	Friable, “fluffy” aspect	large bladder (250-500mL)	narrow bladder neck
110	<50 yo	Male	no relevant history	2 to 3 lesions	Trigone	1-3cm	papillary with a thin pedicle	very large bladder (>500mL)	mid-size prostate (30-60mL)
111	>65 yo	Male	no relevant history	4 to 10 lesions	Ureteric orifice	1-3cm	ill-defined tumour limits	small bladder (<100mL)	large median lobe
112	>65 yo	Male	no relevant history	4 to 10 lesions	Bladder neck	Large denudated area suspicious of CIS (>5c	with abnormal mucosa extending beyond	thin bladder wall	Urethral stricture
113	50-65 yo	Male	no relevant history	over 10 lesions	Prostatic urethra	< 1cm	ill-defined tumour limits	irregular bladder wall, trabeculations	Narrow bladder neck
114	>65 yo	Female	Obese (BMI>30)	Single lesion	Posterior wall	< 1cm	Friable, “fluffy” aspect	normal bladder (100-250mL)	no relevant features
115	>65 yo	Female	no relevant history	2 to 3 lesions	Ureteric orifice	< 1cm	papillary with a stout, large pedicle	normal bladder (100-250mL)	no relevant features
116	50-65 yo	Male	Hx of bleeding disorder or	2 to 3 lesions	Posterior wall	1-3cm	with large turgescent veins	large bladder (250-500mL)	very large prostate (>90mL)
117	>65 yo	Female	Hx of intravesical chemot	Single lesion	Posterior wall	resection of a TURBT scar (e.g “second look	with abnormal mucosa extending beyond	small bladder (<100mL)	no relevant features
118	<50 yo	Male	no relevant history	4 to 10 lesions	Lateral wall	< 1cm	with abnormal mucosa extending beyond	small bladder (<100mL)	large prostate (60-90mL)
119	>65 yo	Male	Hx of prostatectomy	4 to 10 lesions	Lateral wall	< 1cm	papillary with a stout, large pedicle	thin bladder wall	no relevant features
120	50-65 yo	Male	Hx of hip surgery	4 to 10 lesions	Trigone	>5cm	ill-defined tumour limits	very large bladder (>500mL)	not amenable to the lithotomy position
121	<50 yo	Male	no relevant history	4 to 10 lesions	anticipate obturator jerk	1-3cm	ill-defined tumour limits	bladder hernia	mid-size prostate (30-60mL)
122	>65 yo	Male	no relevant history	2 to 3 lesions	Lateral wall	3-5cm	papillary with a thin pedicle	irregular bladder wall, trabeculations	small prostate (<30mL)
123	50-65 yo	Female	no relevant history	2 to 3 lesions	Trigone	3-5cm	with abnormal mucosa extending beyond	bladder hernia	no relevant features
124	<50 yo	Male	Hx of prostatectomy	Single lesion	Lateral wall	1-3cm	papillary with a thin pedicle	large bladder (250-500mL)	Narrow bladder neck
125	>65 yo	Male	no relevant history	over 10 lesions	Lateral wall	Large micropapillary area (>5cm2)	papillary with a thin pedicle	large bladder (250-500mL)	not amenable to the lithotomy position
126	<50 yo	Male	no relevant history	over 10 lesions	Anterior wall	< 1cm	with large turgescent veins	irregular bladder wall, trabeculations	large median lobe
127	<50 yo	Male	no relevant history	2 to 3 lesions	Trigone	< 1cm	papillary with a thin pedicle	normal bladder (100-250mL)	small prostate (<30mL)
128	50-65 yo	Male	Hx of intravesical chemot	4 to 10 lesions	Lateral wall	< 1cm	Sessile or solid aspect	bladder hernia	large median lobe
129	<50 yo	Female	Hx of hip surgery	2 to 3 lesions	Trigone	3-5cm	Friable, “fluffy” aspect	large bladder (250-500mL)	not amenable to the lithotomy position
130	50-65 yo	Male	no relevant history	over 10 lesions	Prostatic urethra	1-3cm	ill-defined tumour limits	bladder hernia	large prostate (60-90mL)
131	50-65 yo	Male	no relevant history	2 to 3 lesions	inside a diverticulum	1-3cm	Sessile or solid aspect	large bladder (250-500mL)	mid-size prostate (30-60mL)
132	<50 yo	Male	Hx of pelvic radiation	2 to 3 lesions	Lateral wall	>5cm	with large turgescent veins	normal bladder (100-250mL)	very large prostate (>90mL)
133	50-65 yo	Male	no relevant history	over 10 lesions	Ureteric orifice	>5cm	with large turgescent veins	large bladder (250-500mL)	Urethral stricture
134	>65 yo	Male	no relevant history	4 to 10 lesions	Anterior wall	Large denudated area suspicious of CIS (>5c	with large turgescent veins	large bladder (250-500mL)	small prostate (<30mL)
135	>65 yo	Female	Hx of recent (<3 months)	over 10 lesions	Lateral wall	< 1cm	papillary with a thin pedicle	small bladder (<100mL)	no relevant features
136	<50 yo	Female	Obese (BMI>30)	over 10 lesions	Posterior wall	1-3cm	Sessile or solid aspect	large bladder (250-500mL)	not amenable to the lithotomy position
137	>65 yo	Male	no relevant history	Single lesion	Lateral wall	1-3cm	papillary with a stout, large pedicle	irregular bladder wall, trabeculations	not amenable to the lithotomy position
138	50-65 yo	Male	no relevant history	4 to 10 lesions	Anterior wall	< 1cm	with large turgescent veins	very large bladder (>500mL)	very large prostate (>90mL)
139	50-65 yo	Female	Hx of intravesical chemot	2 to 3 lesions	Bladder neck	>5cm	ill-defined tumour limits	large bladder (250-500mL)	urethral stricture
140	50-65 yo	Male	no relevant history	Single lesion	inside a diverticulum	< 1cm	ill-defined tumour limits	bladder hernia	large prostate (60-90mL)
141	>65 yo	Male	no relevant history	Single lesion	Ureteric orifice	Large micropapillary area (>5cm2)	ill-defined tumour limits	thin bladder wall	large median lobe
142	>65 yo	Male	Obese (BMI>30)	2 to 3 lesions	Trigone	1-3cm	ill-defined tumour limits	normal bladder (100-250mL)	small prostate (<30mL)
143	>65 yo	Male	no relevant history	over 10 lesions	Lateral wall	Large micropapillary area (>5cm2)	with large turgescent veins	very large bladder (>500mL)	not amenable to the lithotomy position
144	<50 yo	Male	Obese (BMI>30)	4 to 10 lesions	Bladder neck	Large denudated area suspicious of CIS (>5c	with large turgescent veins	very large bladder (>500mL)	Urethral stricture
145	>65 yo	Male	no relevant history	Single lesion	Posterior wall	Large micropapillary area (>5cm2)	with abnormal mucosa extending beyond	irregular bladder wall, trabeculations	Urethral stricture
146	>65 yo	Female	no relevant history	over 10 lesions	Ureteric orifice	< 1cm	with abnormal mucosa extending beyond	normal bladder (100-250mL)	narrow bladder neck
147	<50 yo	Female	Hx of intravesical BCG	4 to 10 lesions	anticipate obturator jerk	3-5cm	papillary with a stout, large pedicle	very large bladder (>500mL)	urethral stricture
148	<50 yo	Male	no relevant history	over 10 lesions	Ureteric orifice	Large denudated area suspicious of CIS (>5c	with large turgescent veins	very large bladder (>500mL)	large median lobe
149	50-65 yo	Male	Hx of recent (<3 months)	Single lesion	anticipate obturator jerk	resection of a TURBT scar (e.g “second look	Sessile or solid aspect	bladder hernia	large prostate (60-90mL)
150	50-65 yo	Male	no relevant history	2 to 3 lesions	Trigone	3-5cm	papillary with a thin pedicle	large bladder (250-500mL)	very large prostate (>90mL)
